# Size-dependent visible absorption and fast photoluminescence decay dynamics from freestanding strained silicon nanocrystals

**DOI:** 10.1186/1556-276X-6-320

**Published:** 2011-04-11

**Authors:** Soumen Dhara, PK Giri

**Affiliations:** 1 Department of Physics, Indian Institute of Technology Guwahati, Guwahati-781039, India; 2 Centre for Nanotechnology, Indian Institute of Technology Guwahati, Guwahati-781039, India

## Abstract

In this article, we report on the visible absorption, photoluminescence (PL), and fast PL decay dynamics from freestanding Si nanocrystals (NCs) that are anisotropically strained. Direct evidence of strain-induced dislocations is shown from high-resolution transmission electron microscopy images. Si NCs with sizes in the range of approximately 5-40 nm show size-dependent visible absorption in the range of 575-722 nm, while NCs of average size <10 nm exhibit strong PL emission at 580-585 nm. The PL decay shows an exponential decay in the nanosecond time scale. The Raman scattering studies show non-monotonic shift of the TO phonon modes as a function of size because of competing effect of strain and phonon confinement. Our studies rule out the influence of defects in the PL emission, and we propose that owing to the combined effect of strain and quantum confinement, the strained Si NCs exhibit direct band gap-like behavior.

## Introduction

The discovery of unusual quantum-induced electronic properties, including photoluminescence (PL), from Si nanocrystals (NCs) has aroused huge scientific interest on Si nanostructures [1-3]. The origin of the PL in the Si NCs is still being debated because of difficulty in isolating the contributions of quantum confinement, surface states and embedding matrix have on the band structure in these materials [4,5]. In general, Si NCs are embedded in other materials with different elastic constants and lattice parameters. In such a case, owing to the lattice mismatch, the consequent elastic strain is known to impact their properties [6]. Lioudakis et al. [7] investigated the role of Si NCs size and distortion at the grain boundary on the enhanced optical properties of the nanocrystalline Si film with the thickness range of 5-30 nm using spectroscopic ellipsometry. They showed that, in the strong confinement regime (≤2 nm), the increase in interaction between fundamental band states and surface states due to distortion results in pinning up of absorption bands. Lyons et al. [8] studied the tailoring of the optical properties of embedded Si nanowires through strain. Thean and Leburton studied the strain effect in large Si NCs (10 nm) embedded in SiO_2 _and showed that coupling between the Si NCs and the strain potential can enhance the confinement [9]. Thus, one would expect an enhanced quantum confinement effect resulting in increased band gap for strained Si NCs as compared with the unstrained Si NCs. Several authors have studied the role of strain and quantum confinement on the optical emission of semiconductor NCs, including Si NCs embedded in a SiO_2 _matrix [9,10] and Ge NCs embedded in SiO_2 _[11]. While these studies find evident strain effects on the band gap, to our knowledge, no study has focused on the coupled effects of size and strain on freestanding Si NCs. Recent reports on the visible PL from freestanding core-shell Si quantum dots provide evidence of quantum confinement-induced, widened band gap-related transitions, and oxide-associated interface-state-related transitions [12,13]. However, the effect of lattice strain in the observed PL emission had been completely ignored in these studies.

In this letter, we investigated the strain evolution and resulting changes in the optical properties of the freestanding strained Si NCs with size down to approximately 5 nm. Microstructure of the Si NCs is studied by high-resolution transmission electron microscopy (HRTEM). Si NCs size and anisotropy in strain are calculated from detailed analysis of X-ray diffraction (XRD) line profile. The optical properties are studied using UV-Vis-NIR absorption, PL, and Raman measurements. Mechanisms of visible PL and fast PL decay dynamics are discussed in the framework of anisotropic strain and confinement effects on Si NCs.

## Experimental

Commercial high purity Si powder (particle size approximately 75 μm, Sigma-Aldrich, Germany) was ball-milled at 450 rpm for a duration of 2-40 h in a zirconia vial (Retsch, PM100) under atmospheric condition using small zirconium oxide balls at a weight ratio of 20:1 for Si powder. Very fine Si NCs with few nanometer sizes obtained after every 2, 5, 10, 20, 30, and 40 h of ball-milling were studied. These samples are named as Si-2, Si-5, Si-10, Si-20, Si-30, and Si-40, respectively. The size, strain, microstructure, and related dislocation density were calculated from powder XRD (Seifert 3003 T/T) pattern and verified by HRTEM (JEOL, JEM-2100) imaging. For careful determination of average NCs size, internal lattice strain, and dislocation density, XRD data were collected at a slow rate at of 0.0025°/s. The UV-Vis-NIR absorption spectra of all the samples were recorded using a commercial spectrometer (Shimadzu 3010PC) at room temperature. Steady-state PL (Thermo Spectronic, AB2) measurements were performed using a Xenon lamp source at different excitation wavelengths and also with a 488-nm Ar laser as an excitation source. The PL decay measurements were performed with 475-nm laser excitation using a commercial fluorimeter (Edinburgh, LifeSpecII,) with time resolution better than 50 ps. Raman scattering measurement was carried out with a 488-nm Ar^+ ^laser excitation using a micro-Raman spectrometer (Jobin Yvon, LabRAM HR-800) equipped with a liquid nitrogen-cooled charge-coupled device detector.

## Results and discussion

**Figure 1:**
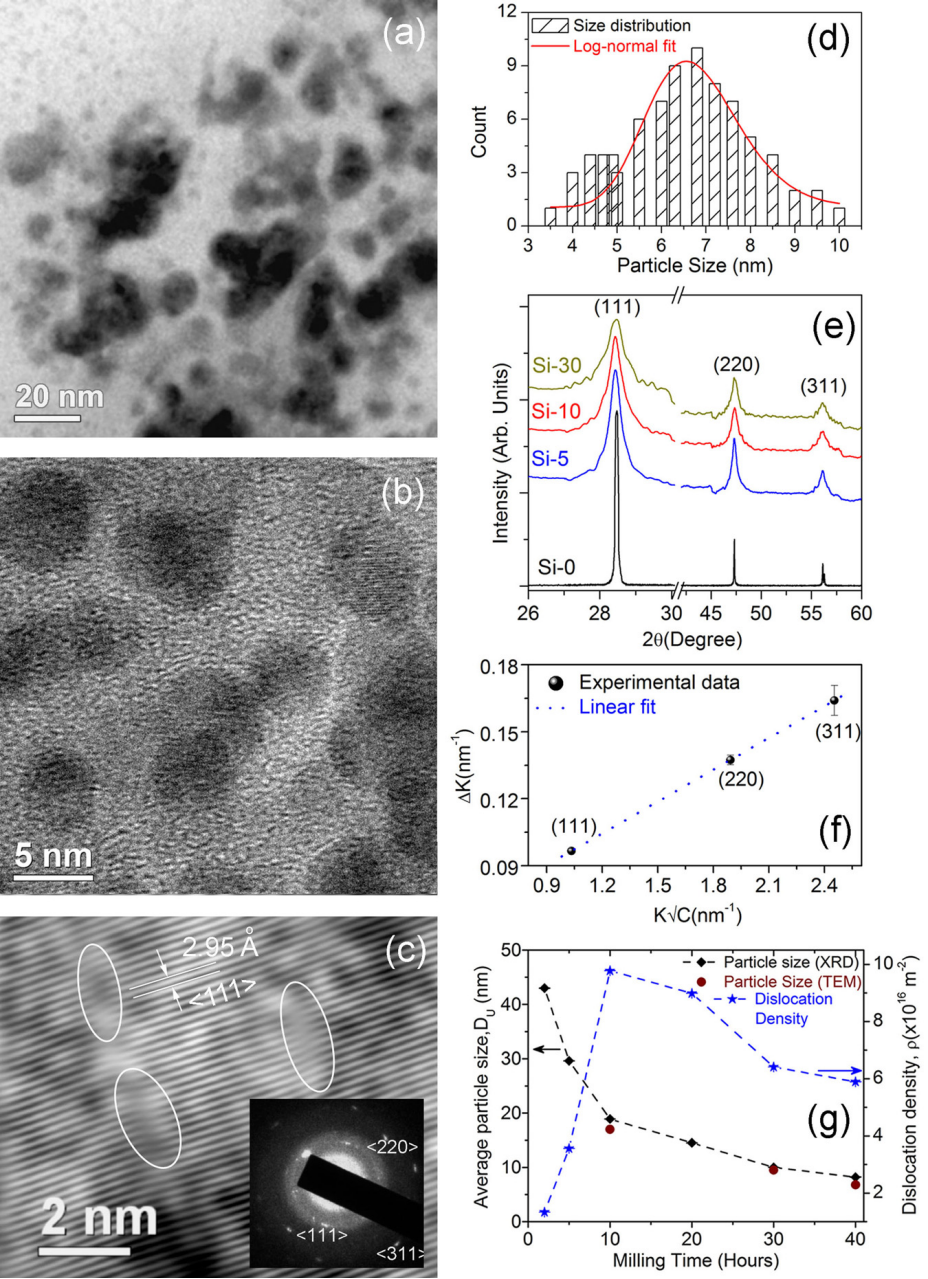
**HRTEM images and XRD spectra of the freestanding Si NCs**. **(a, b) **HRTEM image of the freestanding Si NCs for Si-30 and Si-40, respectively. **(c) **HRTEM lattice image of Si-10 NCs showing distorted lattice (regions marked with oval ring) due to the presence of compressive strain. Inset shows the SAED pattern of the Si-NCs. **(d) **The histogram of size distribution of NCs in Si-40. Lognormal fitting (red line) to the size distribution shows an average size of 6.8 nm. **(e) **The XRD spectra of the Si NCs with different durations of milling and unmilled Si powder. **(f) **Ungar and Borbely plot for Si-10. The linear fit to the experimental data is shown with dotted line. **(g) **Evolution of size and dislocation density with the milling time for Si NCs as calculated from the above plot. For comparison, sizes obtained from HRTEM images are also shown with solid circles. The error bars are too small to be seen in the graph.

Owing to the high speed grinding, substantial size reduction occurs after 2-40 h of milling. The sample milled for 30 h shows the Si NCs with sizes 7-14 nm, and most of the NCs are not purely spherical (Figure 1a). The shape transformation is due to the development of anisotropic lattice strain in the Si NCs, as seen from HRTEM images and XRD studies. After another 10 h of milling, we obtained nearly spherical Si NCs with sizes in the range of 3.5-10 nm, as shown in the HRTEM image in Figure 1b. These NCs are single crystalline, as indicated by clear lattice fringes (Figure 1c) and small area electron diffraction pattern (inset of Figure 1c). In Si-10, lattice strain (distortion) caused by dislocations is clearly observed in the region marked with oval ring in Figure 1c. Careful analysis shows that the interplanar spacing *d*_<111>_decreases from 3.13 to 2.95 Å because of size reduction implying a compressive strain developed during milling. Figure 1d shows the histogram of the size distribution for Si-40. It is noted that a lognormal fitting to size distribution yields an average NC size of 6.8 nm, while many NCs have diameter below 6 nm. Similarly, Si-30 shows an average NC size of approximately 10 nm.

During the milling process, owing to deformation, strain is expected in the as-prepared Si NCs. The XRD spectra of the freestanding Si NCs obtained after different durations of milling are shown in Figure 1e along with the XRD pattern of the unmilled Si powder (Si-0). All the milled Si NCs show strong characteristic XRD peaks for the Si (111), (220), and (311) planes, which confirms high crystalline nature. Our XRD studies on the milled NCs indeed show large broadening in the XRD pattern because of the size reduction and development of strain. To isolate the contribution of strain and size in the observed broadening, XRD line profile analysis is performed following the method of Ungar and Borbely [14]. According to this method, individual contribution of size and strain to the line broadening can be expressed as(1)

where Δ*K *= (2β cos *θ*_B_)/λ, *β *is the FWHM (in radians) of the Bragg reflections; *θ *is the Bragg angle of the analyzed peak; *λ *is the wavelength of X-rays; *D*_U _is the average crystallite size; *K *= 2sin *θ*_B_*/λ*; *e *is the strain; and *C *is the dislocation contrast factor, respectively. Details of the calculation of size and strain evolution in Si NCs sizes and strain are reported elsewhere [15]. Our analysis shows clear evidence for anisotropic strain in these NCs. If dislocations are the main contributors to strain (as evidenced from HRTEM image), then the average crystallite size and dislocation density are calculated from a linear fit to Equation 1 (see Figure 1f). The factor C explicitly incorporates the elastic anisotropy of lattice strain. Efficacy of this method has been demonstrated for several systems, including freestanding Ge NCs [16]. Analysis shows that screw-type dislocations are main contributors to the strain in Si NCs. The evolution of crystallite size and dislocation density (strain) as a function of milling time is shown in Figure 1g. For comparison, size obtained from the HRTEM analysis is also shown in Figure 1g. The sizes obtained from both theses analyses are in close agreement. XRD analysis shows that the average NC size monotonically goes down from 43 to 8.2 nm as the milling time increases from 2 to 40 h. On the other hand, the strain/dislocation density first increases up to 10 h of milling and then it slowly decrease for higher milling time. This can be explained as follows: during milling, the strain and dislocations first develop; however, for prolonged milling when the dislocation density is high, the crystal breaks up along the slip plane and thus produces smaller sized NCs. In this way, strain is partly released for a prolonged milling time [15].

**Figure 2:**
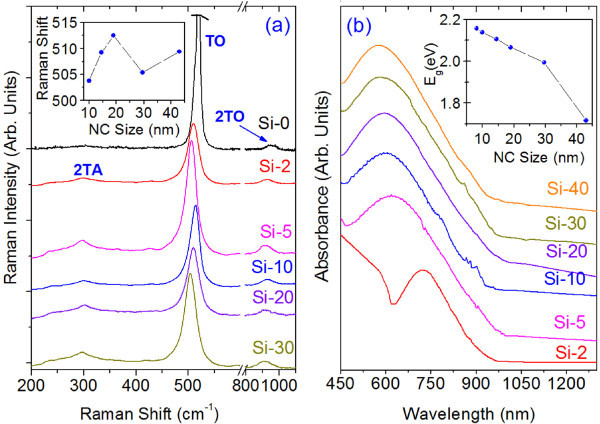
**Micro-Raman and UV-Vis-NIR spectra of the freestanding Si NCs**. **(a) **Micro-Raman spectra of different size Si NCs. Inset shows the plot of Raman shift of TO modes as a function NC size. **(b) **UV-Vis-NIR absorption spectra of the different size Si-NCs. Inset shows the band gap (*E*_g_) calculated from the absorption spectra as a function of NC size.

The presence of lattice strain and possible phonon confinement in Si NCs were further studied by micro-Raman analysis, and the results are shown in Figure 2a. The pristine Si powder exhibits a sharp peak at 520 cm^-1 ^associated with the transverse optical (TO) phonon mode and second-order modes at 300 and 960 cm^-1 ^corresponding to 2TA and 2TO modes, respectively. A plot of Raman shift of TO phonon modes as a function of NC size is shown as inset of Figure 2a. It is noted that the TO modes for different sized NCs show large red shift (from 520 cm^-1 ^down to 503.8 cm^-1^) and line shape broadening (from 10.2 up to 26.6 cm^-1^) with respect to pristine Si powder. Such a large red shift cannot be accounted for phonon confinement effect, as the Si NC sizes are quite large here, especially in Si-2 and Si-5. Thus, the red shift is primarily caused by the local heating of the Si NCs during Raman measurement that uses a 488-nm laser excitation at a sample power of approximately 0.9 mW. Owing to poor thermal conduction in freestanding Si NCs, local heating is expected to be significant. It has been reported that because of local heating by laser excitation, TO phonon modes shows a significant red shift for Si nanowires [17] and Si nanogranular film [18]. Heating effect is expected to increase with decreasing NC size. Possible contribution of ultrathin native oxide layer on Si NCs to the red shift cannot be ruled out, as we observe even higher red shifts for these NCs when oxidized during prolonged storage in air ambient. It is noted that with increasing milling time (up to 10 h), the strain first increases (see Figure 1g) along with size reduction. Owing to the presence of a large compressive strain (as evidenced from HRTEM analysis), one would expect a blue shift in the TO mode that is consistent with our observation in Si-10, as it shows the maximum strain. Therefore, from Si-2 to Si-20, the observed red shifts are due to the competitive effect of local heating and compressive strain in the lattice, as both increase with the size reduction. As there is a sudden increase in the compressive strain in Si-10, the blue shift due to the compressive strain is dominant over heating-induced red shift, this results in a blue shift compared with Si-5. In the case of Si-20, with size reduction, heating-induced red shift increased but, owing to strain relaxation, blue shift is decreased, which effectively results in a red shift. However, in Si-30, owing to further reduction in size as well as reduced strain, a large red shift is observed. Apparently, a higher intensity Raman peak in Si-30 also implies a lower strain in the NCs. In comparison to Si-20, in Si-30 and Si-40, the phonon confinement effect may contribute considerably to the observed higher red shift. Thus, despite the influence of local heating, Raman spectra clearly show the compressive strain effect in all NCs, while the phonon confinement effect is observed for NCs in Si-30 and Si-40. It appears that at sizes <10 nm, the strained Si NCs may be exhibiting enhanced electron and phonon confinement effect because of combined effect of strain and quantum confinement. This is consistent with the theoretical prediction by Thean and Leburton [9], which showed an enhanced confinement effect on the strained Si NCs of large size (10 nm). Earlier, similar quantum confinement-related band structure modification has been observed by Lioudakis et al. [19] from nanocrystalline Si film (approximately 10 nm). Such enhanced confinement effect can be probed by optical absorption and PL emission from the strained Si NCs. Alonso et al. [20] and Lioudakis et al. [21] provided evidence for quantum confinement effect on inter-band optical transitions in SiO_2 _embedded Si NCs for diameter below 6 nm. Owing to the possible presence of native oxide layer on Si NCs, core diameter of the NCs may be actually smaller than the diameter observed in HRTEM. It is noted that despite the presence of anisotropic strain, no splitting of the LO-TO mode was observed in this study perhaps because of random orientation and size distribution of the Si NCs that essentially broaden the Raman spectra.

Figure 2b shows the absorption spectra of the strained Si NCs showing a strong absorption peak in the green portion of the visible spectrum. A systematic blue shift in absorption peak is observed with decrease in NCs sizes, which is an indication of band gap widening of the NCs. In case of Si-30 and Si-40, most of the Si NCs sizes are of the order of Bohr diameter (approximately 9.8 nm) of electron in Si, where a quantum confinement effect is expected [20,22]. However, we observed blue shifts for all the NCs with sizes ranging from 4 to 40 nm. Though the as-prepared Si NCs are likely to have an ultrathin native oxide layer, the size-dependent absorption and low energy of the absorption peak cannot be ascribed to oxide layer or the oxygen-related-defect states. Therefore, strain-induced enhanced quantum confinement effect may play an important role for the band gap widening (as shown in inset of Figure 2b). Thean and Leburton [9] theoretically calculated the band gap widening of Si NCs as a function of strain and showed that the coupling between the Si NCs geometry and the symmetry generated by the strain potential can enhance the confinement in the quantum dot and can lift the degeneracy of the conduction band valleys for nonspherically symmetric NCs. In the present case, many of the anisotropically strained Si NCs are nonspherical (see Figure 1b). Hence, lattice strain may have caused enhanced confinement effect that gave rise to the widening of band gap in these Si NCs, as evident from the absorption spectra. Hadjisavvas and Kelires [23] have also theoretically shown the influence of strain and deformation to the pinning of the fundamental energy band gap of the Si NCs embedded in amorphous oxide matrix.

**Figure 3:**
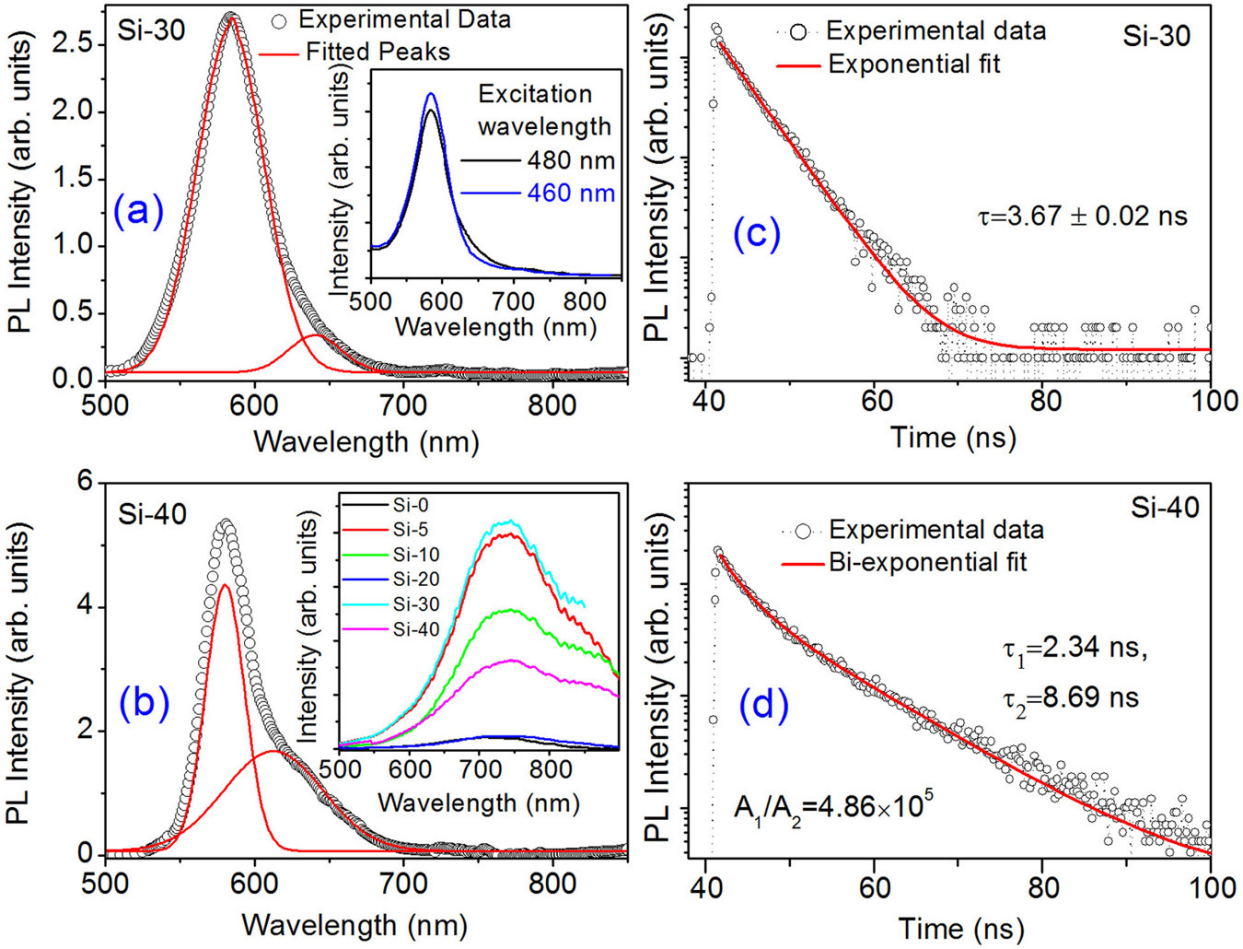
**Steady-state PL and PL decay dynamics spectra of the freestanding Si NCs**. **(a) **Room temperature PL spectra of as-prepared Si-30. Two Gaussian peaks are fitted (solid line) to the experimental data (symbol). Inset shows PL spectra for two different excitation wavelengths. **(b) **PL spectra for as-prepared Si-40. Fitted peaks are shown with solid line. The inset shows the broad PL spectra of different samples after room temperature prolonged oxidation of the Si-NCs. **(c, d) **The PL decay dynamics (intensity in logarithmic scale) of Si-NCs for Si-30 and Si-40 at emission wavelengths 585 and 580 nm, respectively. The exponential fits are shown with solid line in each case.

The Si NCs in Si-30 and Si-40 show strong PL emission in the visible region, which requires fitting of two Gaussian peaks, as shown in Figure 3a,b. The centers of the two peaks are located at 585 and 640 nm for Si-30, and 580 and 613 nm for Si-40, respectively. The emission peaks for the Si-40 is blue shifted, and the peak intensity is also enhanced compared with Si-30. It is noted that no visible PL emission was detected from the as-prepared NCs in Si-5, Si-10, and Si-20, all of which have average NC sizes above 10 nm. However, after prolonged storage in ambient air that causes a thicker oxide layer on the Si NCs, we observe a broad PL emission band at approximately 750 nm from all the samples excited with 488-nm laser, as shown in inset of Figure 3b. As the PL data shown in Figure 3a,b are recorded soon after the milling process, native oxide layer thickness is too small to contribute toward any discernable peak at approximately 750 nm in Figure 3a,b. The approximately 750-nm broad peak is attributed to oxygen-related-defect states in surface oxide layer [13].

We note that 585-nm peak is very strong as compared to the 640-nm peak in Si-30 and this shows a blue shift and higher intensity peak at 580 nm for Si-40, because of to size reduction. Further, the 585-nm peak in Si-30 is found to be completely independent of the excitation wavelength, whereas the 640-nm peak shifts to lower wavelength (higher energy) of 629 nm when excited at a lower wavelength, as shown in the inset of Figure 3a. This excitation energy dependence of the 640-nm peak strongly indicates its origin as surface/interface defect-related states. On the other hand, 585-nm peak cannot originate from defect-related state. Wilcoxon et al. [24] reported on the appearance of PL peaks in the range 1.8-3.6 eV for different sizes of Si NCs. The intense violet peak was assigned to direct electron-hole recombination, whereas the less intense PL peak (approximately 600 nm) was attributed to the surface states and phonon-assisted recombination. Lioudakis et al. [7] showed that L-point indirect gap of nanocrystalline Si film increases monotonically with decreasing film thickness down to 5 nm, as exactly predicted from the quantum confinement theory. Since the excitation wavelength of 460 nm is above the L-point gap (indirect) of Si-30, phonon-assisted recombination is likely to contribute to the 640-nm PL peak in Si-30. Similarly, Ray et al. [13] ascribed the PL bands at approximately 600 and 750 nm from core-shell Si/SiO_2 _quantum dots to oxide-related interface defect states. Therefore, phonon-assisted recombination is most likely to be responsible for the low intensity peak at 613-640 nm. However, the strong emission at 580-585 nm cannot arise from such a process. It is noted that in the literature, less intense PL peak at around approximately 600 nm from Si NCs is generally attributed to surface states only for very small NCs (<3-4 nm).

PL excitation measurements for Si-30 and Si-40 at their corresponding emission wavelengths (585/580 nm) show that Stokes shift is very insignificant (approximately 0.067 eV). This is also obvious from the relatively close position of the absorption and emission peaks for Si-30 and Si-40. Such a small shift again rules out the involvement of defects or interface states being responsible for the observed PL. This may indicate a direct transition from valence band to conduction band in the Si NCs. Further, if the interface defects or oxide layer contribute to the 585 nm PL, then one would expect this band from all the samples that show absorption in the visible region, which is contrary to the observation. Therefore, strain-induced enhanced quantum confinement may responsible for the observed PL band at 580-585 nm.

To further understand the nature of transition, we studied the PL decay dynamics of the observed band at 580/585 nm (Figure 3c,d). For Si-30, the decay profile fits to a single exponential decay with time constant τ_1 _= 3.67 ns, while for Si-40, it fits to a bi-exponential decay with time constants τ_1 _= 2.34 ns, τ_2 _= 8.69 ns. It is noted that for Si-40, amplitude of the fast decay component (τ_1_) is about six orders of magnitude higher than that of the slow component (τ_2_). This is consistent with the steady-state PL spectra that show a very strong peak at 580 nm as compared to the weak band at 613 nm. Further, reduction in τ_1 _from 3.67 to 2.34 ns with size reduction in Si-40 is consistent with the quantum confinement effect, and this minimizes the possibility of the fast decay dynamics to be attributed to defect states. Most of the reported PL decay behavior of Si NCs has lifetime values in the range of microseconds to a few milliseconds and the NCs are usually embedded in SiO_2 _matrix [25-28], while some studies reported decay in the nanosecond time scale [29,30]. In the present case, Si NCs are freestanding with minimum influence of native oxide layer, and emission is monitored specifically at 580/585 nm. Since the 580/585-nm PL band does not originate from defects, the observed properties are believed to be intrinsic to the strained Si NCs core. We believe that this fast decay dynamics is a signature of formation of quasi-direct energy bands in the band structure of the Si NCs, since in the case of quasi-direct nature of transition the electron-hole recombination process is very fast [22]. However, possible contribution of non-radiative decay channel in the observed fast PL decay cannot be fully ruled out. Othonos et al. [31] showed that surface-related states in the oxidized Si NCs can enhance the carrier relaxation process and Auger recombination does not play a significant role even in small NCs. It may be noted that this study deals with Si NCs that are freestanding and not oxidized (intentionally).

Based on these observations and recent reports [12,13], we are inclined to suggest that dominant transition involving strain-induced, enhanced quantum confinement-related, widened band gap states are responsible for the distinct visible absorption and an intense visible PL at 580-585 nm from the freestanding Si NCs. While the absorption/photoexcitation of carriers is certainly a band-to-band transition process, higher wavelength emission bands are though to be defect mediated. Such transitions can take place via a three-step process: (i) creation of electron-hole pairs inside the crystalline core, followed by (ii) nonradiative relaxation of electrons within the band, and (iii) subsequent radiative de-excitation of the electron to the valence band of the core. As the Stokes shift is very small for the 580/585 nm band, the thermal relaxation loss is very small. Hence, the photoexcited carriers in this case are not at all relaxing at the band edge or at the interface states, they are possibly relaxing within the band. The higher size as-prepared Si NCs did not exhibit the approximately 585-nm PL band partly because of the absence of quantum confinement effect and partly because of the presence of high density of dislocations, as evident from Figure 1. These dislocations usually quench the PL, and hence no PL signal was detected.

## Conclusions

In conclusion, we synthesized anisotropically strained freestanding Si NCs with sizes approximately 5-42 nm that are freestanding and studied the optical absorption and PL emission from these NCs as a function of its size. The Raman studies show that besides the local heating effect that causes a substantial downshift, TO modes upshift because of compressive strain in all the NCs, while the phonon confinement-induced downshift is observed for NCs with average size below 10 nm. The observed enhanced visible absorption and the systematic blue shift in absorption peak with size reduction are believed to be caused by the combined effect of lattice strain and quantum confinement effects. Size-dependent strong PL band at 585 nm and the fast PL decay dynamics for this band are believed to be caused by the quasi-direct energy bands in the strained Si NCs. Role of defects in the 585-nm PL emission was ruled out. These results imply that strain engineering of Si NCs would enable tunable visible light emission and fast-switching light-emitting devices.

## Abbreviations

HRTEM: high-resolution transmission electron microscopy; NCs: nanocrystals; PL: photoluminescence; TO: transverse optical; UV-Vis-NIR: ultraviolet-visible-near infrared; XRD: X-ray diffraction.

## Competing interests

The authors declare that they have no competing interests.

## Authors' contributions

SD carried out all the experiments and analyses of the data. SD and PKG together interpreted the results and prepared the manuscript.
